# A Coarse-to-Fine Framework for Mid-Radiotherapy Head and Neck Cancer MRI Segmentation

**DOI:** 10.1007/978-3-031-83274-1_11

**Published:** 2025-03-03

**Authors:** Jing Ni, Qiulei Yao, Yanfei Liu, Haikun Qi

**Affiliations:** 1 ShanghaiTech University, Shanghai, China; 2 Shenzhen United Imaging Research Institute of Innovative Medical Equipment, Shenzhen, China

**Keywords:** Head and neck cancer, 3D segmentation, Coarse-to-Fine, Magnetic Resonance Image

## Abstract

Radiotherapy is the preferred treatment modality for head and neck cancer (HNC). During the treatment, adaptive radiation therapy (ART) technology is commonly employed to account for changes in target volume and alterations in patient anatomy. This adaptability ensures that treatment remains precise and effective despite these physiological variations. Magnetic resonance imaging (MRI) provides higher-resolution soft tissue images, making it valuable in target delineation of HNC treatment. The delineation in ART should adhere to the same principles as those used in the initial delineation. Consequently, the contouring performed on MR images during ART should reference the earlier delineations for consistency and accuracy. To address this, we proposed a coarse-to-fine cascade framework based on 3D U-Net to segment mid-radiotherapy HNC from T2-weighted MRI. The model consists of two interconnected components: a coarse segmentation network and a fine segmentation network, both sharing the same architecture. In the coarse segmentation phase, different forms of prior information were used as input, including dilated pre-radiotherapy masks. In the fine segmentation phase, a resampling operation based on a bounding box focuses on the region of interest, refining the prediction with the mid-radiotherapy image to achieve the final segmentation. In our experiment, the final results were achieved with an aggregated Dice Similarity Coefficient (DSC) of 0.562, indicating that the prior information plays a crucial role in enhancing segmentation accuracy. (Team name: TNL_skd)

## 1 Introduction

Head and neck cancer (HNC) is among the most common types of cancer globally; almost 880,000 patients are diagnosed every year [1]. Radiation therapy plays an important role in HNC treatment, but it relies heavily on the accuracy of delineation. A precise manual contouring process of a HNC patient often takes the clinician lots of time and the average reported time taken from 2.7 to 3.0h [2], therefore an effective auto segmentation method is necessary. Due to the higher soft tissue resolution of magnetic resonance images compared to computed tomography images in the head and neck region, magnetic resonance guided radiotherapy (MRgRT) has become increasingly popular in HNC treatment. However, MR-guided ART may require multiple contouring for one patient, making the need for automatic contouring urgent.

In recent years, an increasing number of research has the advantage of deep learning in medical image segmentation [3]. Using positron emission tomography (PET) and computed tomography (CT) to segment head and neck cancer was explored in the HECTOR challenge, and the algorithms from participants showed great results [4–6]. Notably, there is also a growing interest in utilizing MRI for this purpose. Schouten et al. [7] introduced an automated segmentation pipeline for head and neck squamous cell cancer using a multi-view convolutional neural network (MV-CNN) with multi-modal MRI sequences, achieving moderate results. Bielak et al.’s [8] method utilizes multiple MRI contrasts at different time points to train CNNs for lesion segmentation in head and neck cancer. The contribution of each contrast is assessed by comparing a reference CNN with all contrasts to CNNs where one input channel is excluded. A method proposed by Korte et al. involved the development of three convolutional neural network-based auto-segmentation architectures [9]. Their results also demonstrated improved geometric accuracy in the segmentation process. However, these methods, which directly delineate MR images, are limited in their ability to account for inherent structural and spatial changes that may occur during treatment. This presents a challenge for ART, where reference to initial images or contours is necessary for accurate treatment adaptation. Wahid et al. [10] proposed utilizing multiparametric MRI for oropharyngeal cancer primary gross tumor volume auto-segmentation, leveraging additional channel combinations to improve segmentation performance. Their work highlights the importance of incorporating multiple MRI contrasts to achieve more robust segmentation.

Due to the lack of publicly available datasets for evaluating model performance in this area, the HNTS-MRG2024 has been organized, offering a platform to assess and explore adaptive radiotherapy (ART) in real-world clinical scenarios.

**Fig. 1.**
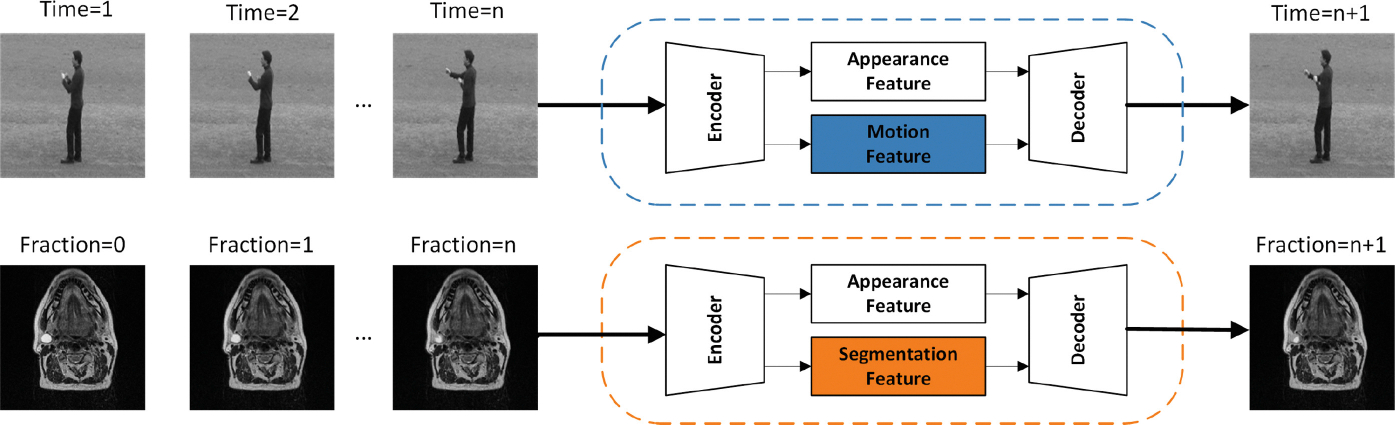
The workflows of ART and future frame prediction.

ART has similarities with future frame prediction, and both techniques rely heavily on prior information to guide future outcomes (Fig. 1). In ART, previous CT or MRI scans provide a reference for adjusting treatment plans when the patient’s anatomy changes. In the same way, during future frame prediction, past frames are used to predict subsequent frames in natural images. Inspired by Z. Gao et al. [11], they proposed a simpler yet effective CNN model for video prediction and achieved state-of-the-art results without introducing any complex modules, strategies, and tricks. Similarly, UNet3D-based next-frame prediction models have also been explored [12], and they have achieved better performance compared to CNN-LSTM and Convolutional LSTMs. In the ref-based segmentation, we can consider it as a combination of one-frame prediction and segmentation.

Our work focuses on task 2 mid-radiotherapy segmentation of the HNTS-MRG2024 challenge. We present an end-to-end UNet-based model that utilizes prior knowledge, specifically through pre-radiotherapy masks, to improve segmentation performance in adaptive radiotherapy. To assess the impact of varying levels of prior information, we conducted three comparative experiments. For the first, no prior information was incorporated. In the second experiment, pre-radiotherapy masks were used as the only source of prior information. In contrast, the third experiment combined both pre-radiotherapy masks and pre-radiotherapy images to evaluate their joint effect on segmentation accuracy.

## 2 Method

### 2.1 Dataset

The HNTS-MRG 2024 challenge training dataset contains 150 patients. The dataset consists of T2-weighted (T2w) anatomical sequences of the head and neck region, collected at the University of Texas MD Anderson Cancer Center. The dataset includes both fat-suppressed and non-fat-suppressed images, with all patients being immobilized during the scans. The mask includes three categories: background, primary gross tumor volumes (GTVp), and metastatic lymph nodes (GTVn). For each case, there are 3 sets of images, including the original pre-radiotherapy T2w MRI, the original mid-radiotherapy T2w MRI, and the registered pre-radiotherapy T2w MRI. Corresponding segmentation masks are also provided for each of these, including the pre-RT segmentation mask, the mid-radiotherapy segmentation, and the registered pre-radiotherapy segmentation mask.

### 2.2 Data Preprocessing

#### Normalization.

In the data preprocessing stage, we used the registered pre-images and masks provided by the official dataset for all 150 training cases. For each image, we selected the area corresponding to the mask and calculated the 0.5th and 99.5th percentiles within this region. These calculated low and high values were then applied to clip the entire image. After that, we used Z-score normalization.

#### Image Cropping.

In the pre-phase, the maximum *xy* dimensions, referring to the cross-sectional plane, are 768 × 768, and the minimum is 512 × 512, with the maximum z-plane being 162. In the mid-radiotherapy, the maximum *xy* dimensions are 768 × 768, and the minimum is 480 × 480, with the maximum z-plane being 168. Additionally, the majority of images have a resolution of 0.5 × 0.5 × 2 mm, with a shape of 512 × 512 × *z*. To meet the input requirement of the UNet structure, we resampled all images to a fixed size of 336 × 336 × 160 with a resolution of 0.5 × 0.5 × 2 mm.

#### Mask Dilated.

We used pre-radiotherapy masks as prior information to segment the mid-radiotherapy images. To adequately overlap with the mid-radiotherapy mask, the radius of the pre-radiotherapy masks was dilated along the *x*, *y*, and *z* axes. In our experiment, we tested dilation radii ranging from 1 to 10, finally selecting 6 pixels for the *xy*-plane and 3 pixels for the z-plane, represented as (6, 6, 3). This choice was based on the spacing resolution of the imaging data and the practical 3 mm clinical margin used by physicians to account for uncertainties in tumor position and treatment delivery. After applying this dilation, the overlap ratio of the mid-radiotherapy mask reached 99.12% for GTVp and 99.71% for GTVn, calculated across all cases by excluding voxels with zero values. These values represent the mean overlap ratios for individual cases after removing zero-value results from the Statistic. By using dilated pre-radiotherapy masks, we could incorporate prior information to guide the model toward more accurate localization, capturing contextual details that improve segmentation performance.

#### Post Processing.

In the inference stage, post-processing was used to refine the results and improve segmentation accuracy. We mainly used connected component analysis and region merging. Connected component analysis was applied to identify different regions, while region merging helped ensure accurate segmentation boundaries and removed smaller irrelevant components. In some cases, the segmentation results may show an overlap between GTVp and GTVn, which does not happen in the clinical reality.

### 2.3 Network Architecture

**Fig. 2.**
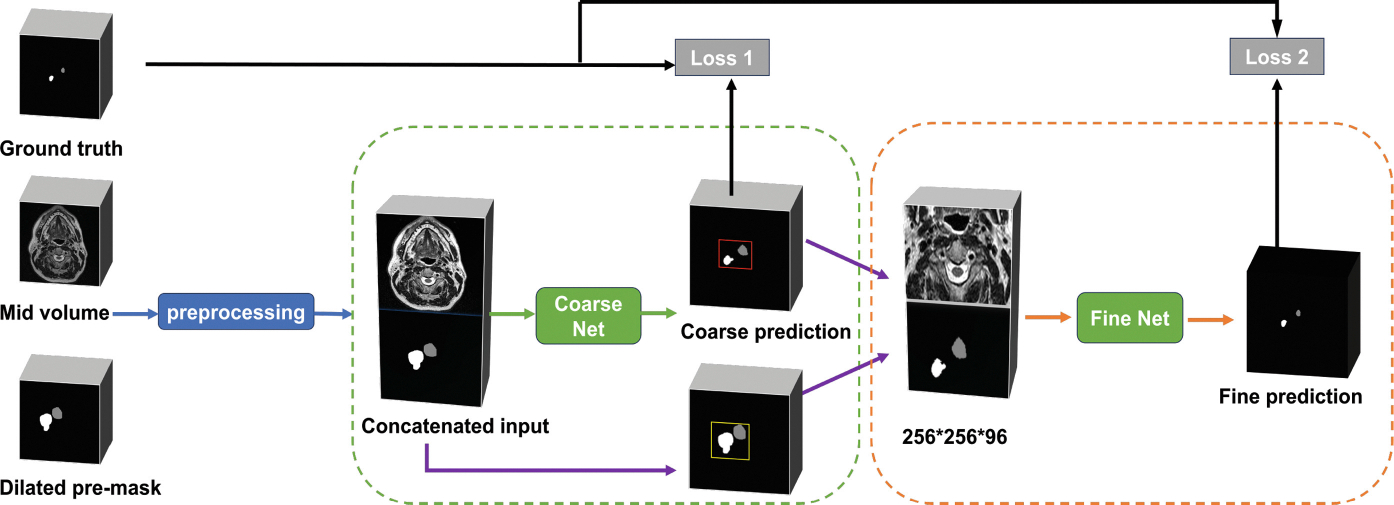
The proposed end-to-end coarse-to-fine segmentation network.

The end-to-end framework is built on encoder-decoder architecture. The network operates on a set of inputs and a corresponding set of outputs. Inspired by the success of Jiang et al. [13] in BraTS Challenge2019 and Sun et al. [5] in HECTOR Challenge2022, we propose an end-to-end coarse-to-fine segmentation network (Fig. 2).

#### Target Location.

Due to the anatomical complexity of the region for head and neck cancer, precise location is the key to delineation. Clinical experience has shown that tumors can both shrink and shift during radiotherapy. GTVp may temporarily swell due to inflammation or the tumor’s biological response, but it often shrinks as treatment progresses [14]. In contrast, GTVn typically decreases in volume throughout radiotherapy. However, positional shifts can occur, influenced by factors such as patient weight loss or changes in positioning during treatment sessions [15]. To accommodate potential tumor movement in ART, we dilated the pre-radiotherapy mask to create a sufficient margin around the tumor. This margin ensures that even with slight anatomical variations during treatment, the tumor remains within the pre-radiotherapy masks, maintaining accurate targeting throughout radiotherapy. We dilated registered pre-radiotherapy masks as prior information and concatenated it with mid-radiotherapy image as input to the model.

#### Coarse Segmentation.

**Fig. 3.**
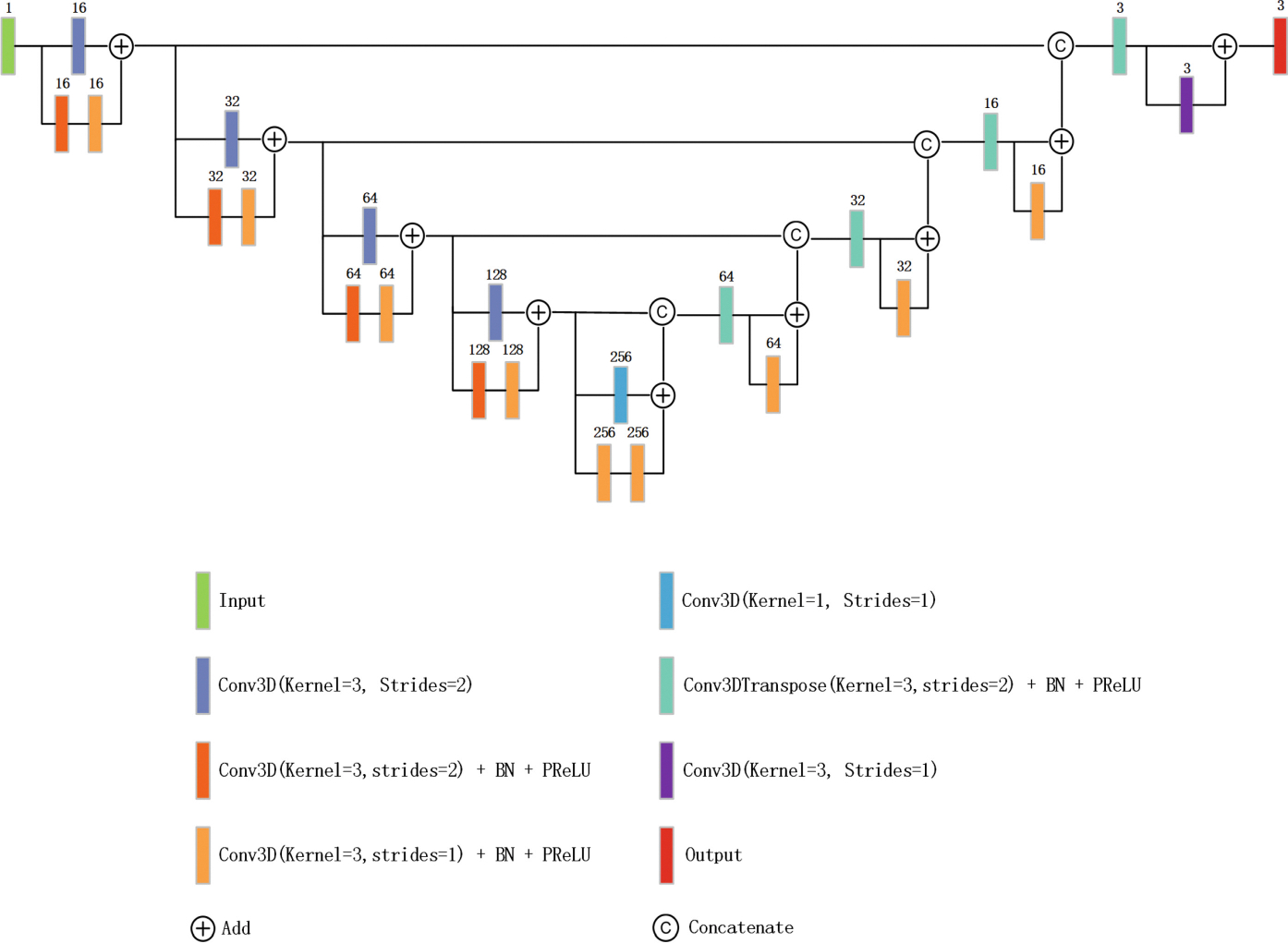
An illustration of the coarse model based on 3D Unet. Here, we show the case with a single-channel input. In our experiments, the input will be adjusted according to different situations

The coarse network is based on the 3D UNet implemented using the MONAI framework (Fig. 3), which adopts an encoder-decoder architecture with symmetric skip connections to preserve spatial information. The encoder path consists of five stages, with channel dimensions progressively increasing from 16, 32, 64, 128 to 256. At each stage in the encoder, residual units with two convolutional layers are used. Those layers with kernel size 3 × 3 × 3 and stride 2 perform downsampling, while batch normalization (BN) and PReLU activation enhance stability. The decoder mirrors the encoder, using transposed convolutions (Conv3DTranspose) to upsample the feature maps. Each upsampling operation doubles the spatial resolution while halving the number of channels. Skip connections between the encoder and decoder are used to concatenate high-resolution details from earlier layers, facilitating better localization in the final segmentation. In the final layer of the decoder, a 1 × 1 × 1 convolution reduces the feature maps to 3 channels, aligning with the output requirement for segmentation classes. This streamlined architecture makes it suitable for segmentation tasks.

#### Bounding Box.

Before performing the fine segmentation, we cropped it again to reduce redundant information. After getting coarse output, we calculated its bounding box relative to the dilated pre-radiotherapy mask. In addition, we respectively calculated the bounding box of the original pre-radiotherapy mask and dilated pre-radiotherapy mask for comparison. The results are shown in Table 1. Therefore, a fixed size of 256 × 256 × 96 was selected, centered on the combined bounding box of the two.

#### Fine Segmentation.

The fine segmentation network architecture is the same as the coarse segmentation network, while they have different inputs. For the fine network, the cropped coarse output was concatenated with the cropped mid-radiotherapy image and used as the input.

### 2.4 Training Details

The training dataset consists of 150 samples, which were split into 80% for training, 10% for validation, and 10% for testing. We used 3-fold cross-validation during training, and the model was trained for 200 epochs in each fold using the DiceCE loss function and the AdamW optimizer [16]. To evaluate the prediction of accuracy, we used the aggregated Dice Similarity Coefficient (DSCagg) [17]. Besides, the CyclicLR schedule was used to adapt the learning rate and prevent overfitting. All the operations were conducted on an NVIDIA GeForce RTX 3090 with 24GB of memory.

#### Data Augmentation.

For data augmentation, we relied on the MONAI platform [18]. We applied zooming, rotation, and affine transformations to both images and masks, while RandAdjustContrastd and RandGaussianNoised were applied only to images. A summary of the training details is as follows (Table 2):

#### Loss Function.

The loss function we chose is DiceCE, which combines the advantages of multi-class dice loss [19] and cross-entropy loss. We applied the loss function to both the coarse and fine networks. Rather than a simple summation, the total loss is weighted dynamically at each stage. In the coarse segmentation (loss 1), the weight decreases with epoch from 0.9 to 0.1, while in the fine segmentation (loss 2), the weight increases from 0.1 to 0.9. To ensure that the coarse segmentation results are as accurate as possible in the early stages of training, we need to implement effective measures. This will contribute to improving the overall performance and effectiveness of subsequent model training.

#### Evaluation Metrics.

The official evaluation metric is DSCagg [20], which minimizes the impact of isolated false negatives or false positives on the overall performance evaluation, ensuring a more robust and balanced assessment.

$${DSC}_{agg}=\frac{2\sum_{i}\;\left|A_{i}\cap B_{i}\right|}{\sum_{i}\;\left|A_{i}\right|+\left|B_{i}\right|}$$

where $A_{i}$ and $B_{i}$ are the ground truth and predictions for image i.

The surface dice similarity coefficient (surface DSC) evaluates the agreement between the segmentation surface and the reference surface [21]. It quantifies the proportion of points on the automatically segmented surface that lie within a specified tolerance distance τ from the reference surface. When evaluating the Surface DSC, it is necessary to define a threshold within which variation is clinically acceptable.

Specifically, a surface S is defined as the boundary of a mask ∂M, with its area represented by $|S|=\int_{S}\;d\sigma$, where σ is a point on the surface. The mapping from this point on the surface to a point in three-dimensional space is represented by ξ(σ). Using this mapping, the border region ${ℬ}_{i}^{\left(\tau \right)}$ for a surface $S_{i}$ at a given tolerance τ is defined as the set of points within a distance of τ from the surface $S_{i}$.


$${ℬ}_{i}^{\left(\tau \right)}=\left\{x\in R^{3}|\exists \sigma \in S_{i},\|x-\xi (\sigma )\|\leq \tau \right\},$$

The surface DSC metric, denoted as $R_{i}^{\left(\tau \right)}$, is then calculated by measuring the overlap of each surface with the border region of the other, normalized by the sum of the surface areas.


$$R_{i,j}^{\left(\tau \right)}=\frac{\left|S_{i}\cap {ℬ}_{j}^{\left(\tau \right)}\right|+\left|S_{j}\cap {ℬ}_{i}^{\left(\tau \right)}\right|}{\left|S_{i}\right|+\left|S_{j}\right|},$$

The Hausdorff Distance (HD) is an important geometric metric used to measure the similarity between two sets of points [22]. It defines the maximum of the minimum distances from points in one set to the closest points in the other set, capturing the worst-case deviation between the two sets. Given two point sets A and B, the Hausdorff Distance H(A,B) is defined as:

H(A,B)=max(h(A,B),h(B,A))

where

$$h(A,B)=\underset{a\in A}{max}\;\underset{b\in B}{min}\;\|a-b\|$$

To exclude unreasonable distances caused by some outliers and maintain overall numerical stability, we use the 95% Hausdorff Distance (95% HD).

## 3 Results

We evaluated the effect of incorporating different types of prior information on the segmentation performance. Specifically, we compared three different input combinations: mid-radiotherapy (mid-RT) image alone, pre-radiotherapy (pre-RT) mask + mid-radiotherapy (mid-RT) image, pre-radiotherapy image + pre-radiotherapy mask + mid-radiotherapy image. Tables 3, 4, 5 presents the segmentation results across these input combinations for GTVp and GTVn. The test process for both models utilized the same architecture and parameters.

The results indicate that using mid-radiotherapy images alone provided a baseline performance while adding the pre-radiotherapy image to the mid-radiotherapy image resulted in noticeable improvements in both GTVp and GTVn segmentation accuracy. Furthermore, using the pre-radiotherapy mask in combination with the mid-radiotherapy image demonstrated the highest mean accuracy in fold 1. Among the results, GTVn consistently outperformed GTVp. These results highlight the positive impact of incorporating prior information to enhance segmentation performance.

We also tested the results of training the coarse and fine networks separately but found it inferior to the end-to-end model. This suggests that the coherence of information is crucial for segmentation tasks.

In the final submission phase, we selected the model using a pre-radiotherapy mask and mid-radiotherapy image. The final test results were: GTVp=0.500, GTVn=0.625, mean=0.562.

## 4 Discussion

The findings suggest that the inclusion of different types of prior information can significantly enhance segmentation performance. The highest performance was achieved when combining pre-radiotherapy masks with mid-radiotherapy images, demonstrating that integrating temporal information along with spatial priors contributes to more robust segmentation. This implies that the model benefits not only from knowing the current anatomy but also from the historical context provided by pre-radiotherapy images, which might help in anticipating deformations or other changes over time.

Our results align with the findings of Wahid et al. [10], where the segmentation of oropharyngeal primary tumors using different types of data (five mpMRI input channels: T2, T1, ADC, Ktrans, Ve) showed that adding all data types did not yield the best performance. Instead, the combination of T1 and T2 achieved the best results. Similarly, in our study, adding different prior information did not always result in better outcomes, but the strategic combination of pre-radiotherapy mask and mid-radiotherapy image was most effective.

Based on the competition results, GTVp was consistently harder to segment than GTVn. Our results for GTVp segmentation were comparable to other participants, which demonstrates that our simple model has a certain level of capability in handling the segmentation of complex structures. However, the GTVn segmentation results showed a larger gap compared to other participants. We attribute this discrepancy to the following reasons: First, we did not use additional datasets, and all training and validation were conducted using the official training set only. Incorporating additional multi-modal training could help the model learn more comprehensive features. Second, we did not introduce modules or techniques to enhance the model’s performance, as our goal was to explore whether a simple model could effectively predict and segment in the ART field. Lastly, our model might have experienced overfitting during training, which could have potentially impacted its ability to generalize effectively.

## 5 Conclusion

In our experiment, the proposed simple cascade model still demonstrates a certain ability to segment and predict the location of mid-radiotherapy cancer targets. This study aims to investigate the impact of prior knowledge on the performance of ART segmentation tasks. We explored how prior knowledge influences segmentation accuracy by incorporating prior information through a dilated pre-radiotherapy mask during the coarse segmentation step and refining the results based on a fixed region determined by the bounding box.

More relevant datasets need to be used for training in future work, allowing the model to learn more effectively. Additionally, incorporating different modalities of data from the same patient as prior knowledge could be investigated to determine if it further improves model performance. Finally, adding small spatial prediction modules to the network might be explored as a potential improvement, which could better serve adaptive radiotherapy by enhancing the accuracy of tumor deformation and positional change predictions.

## Figures and Tables

**Table 1.** Bounding box measurements for pre-radiotherapy mask and dilated pre-radiotherapy mask

Measurement	pre-RT mask	dilated pre-RT mask

*x* range	(27, 247)	(33, 253)
*x* mean	119.74	126.99
*y* range	(23, 198)	(33, 222)
*y* mean	91.12	105.48
*z* range	(5, 69)	(11, 75)
*z* mean	29.13	39.64

**Table 2.** Training methodology and hyper-parameters.

Component	Value

Epochs	200
Batch size	1
Learning rate	3*e*^−5^
Loss function	DiceCE
Optimizer	AdamW
Scheduler	CyclicLR
Data augmentation	RandRotate90d, RandZoomd[0.9, 1.1], RandAffined (scale range[0.1, 0.1, 0.1]), RandAdjustContrastd (gamma[0.9, 1.1]), RandGaussianNoised

**Table 3.** Comparison of Results for Different Inputs using DSCagg

Input	Fold	GTVp	GTVn	Mean

mid-RT image	fold1	0.012	0.292	0.152
	fold2	0.021	0.229	0.125
	fold3	0.003	0.345	0.173

pre-RT mask mid-RT image	fold1	0.536	0.702	**0.619**
	fold2	0.528	0.704	0.616
	fold3	0.477	0.650	0.564

pre-RT image pre-RT mask mid-RT image	fold1	0.426	0.600	0.513
	fold2	0.471	0.745	0.608
	fold3	0.409	0.609	0.509

**Table 4.** Comparison of Results for Different Inputs using surface DSC

Input	Fold	GTVp	GTVn	Mean

mid-RT image	fold1	0.037	0.233	0.135
	fold2	0.107	0.225	0.166
	fold3	0.022	0.238	0.130

pre-RT mask mid-RT image	fold1	0.417	0.613	0.515
	fold2	0.365	0.646	0.506
	fold3	0.391	0.569	0.478

pre-RT image pre-RT mask mid-RT image	fold1	0.420	0.604	0.520
	fold2	0.452	0.667	**0.559**
	fold3	0.431	0.599	0.515

**Table 5.** Comparison of Results for Different Inputs using 95%HD

Input	Fold	GTVp	GTVn	Mean

mid-RT image	fold1	91.685	100.119	96.505
	fold2	59.064	57.349	58.257
	fold3	65.849	58.355	62.102

pre-RT mask mid-RT image	fold1	9.423	9.008	**9.242**
	fold2	16.924	9.282	13.740
	fold3	15.849	11.906	13.972

pre-RT image pre-RT mask mid-RT image	fold1	12.788	35.082	19.476
	fold2	11.460	15.793	13.265
	fold3	17.142	17.054	17.112
